# Application of Shear Wave Sonoelastography in the Differential Diagnosis of Extra- and Intra-Thyroidal Ectopic Thymic Tissue

**DOI:** 10.3390/jcm9123816

**Published:** 2020-11-25

**Authors:** Magdalena Stasiak, Zbigniew Adamczewski, Renata Stawerska, Bartłomiej Stasiak, Andrzej Lewiński

**Affiliations:** 1 Department of Endocrinology and Metabolic Diseases, Polish Mother’s Memorial Hospital—Research Institute, 281/289 Rzgowska St., 93-338 Lodz, Poland; mstasiak33@gmail.com (M.S.); zbigniew.adamczewski@umed.lodz.pl (Z.A.); renata.stawerska@icloud.com (R.S.); 2 Department of Endocrinology and Metabolic Diseases, Medical University of Lodz, 281/289 Rzgowska St., 93-338 Lodz, Poland; 3 Department of Paediatic Endocrinology, Medical University of Lodz, 281/289 Rzgowska St., 93-338 Lodz, Poland; 4 Institute of Information Technology, Lodz University of Technology, 215 Wolczanska St., 90-924 Lodz, Poland; bartlomiej.stasiak@p.lodz.pl

**Keywords:** ectopic thymus, thyroid, shear wave sonoelastography, strain elastography, ultrasound, thyroid cancer, metastatic lymph nodes

## Abstract

The ultrasound (US) pattern of intrathyroidal ectopic thymus (IET) can resemble papillary thyroid carcinoma (PTC) while the extrathyroidal ectopic thymus (EET) can mimic pathological lymph nodes. Recently, the usefulness of strain elastography (SE) was demonstrated in the differential diagnosis, however this method has several limitations. The aim of the current study was to assess the usefulness of shear wave elastography (SWE) in this field. The US, SE, and SWE were performed in 31 children with 53 ectopic thymuses (ETs) and quantitative values of SWE parameters were calculated, so as to generate potential normative values of ET elasticity and of the shear wave ratio (SWR). The mean SWR_IET_ was 0.89 ± 0.21 and the mean shear wave stiffness (SWS) was 7.47 ± 1.93 kPa. The mean SWR_EET_ was 0.84 ± 0.15 and the mean SWS_EET_ was 11.28 ± 2.58 kPa. The results have proven that the stiffness of ETs is lower or equal to the thyroid’s. SWE was demonstrated to be a useful diagnostic method for ET evaluation. Therefore, the application of SWE in ET diagnosis allows more accurate evaluation of ET-like lesions and, in many cases, allows one to avoid invasive procedures, simultaneously providing a precise monitoring method based on combined US and SWE evaluation.

## 1. Introduction

Ultrasonography (US) is an easily accessible, non-invasive diagnostic tool used in neck imaging and a first-line method for the diagnosis of thyroid gland nodules. The increasing availability of the US procedure leads to a new phenomenon of accidental findings of many thyroid and neck lesions in children. On the basis of US and postmortem examinations, the frequency of thyroid nodules in children and young adolescents/young adults was estimated at 1–1.5% and 13%, respectively [1]. The risk of malignancy in thyroid nodules in children is high, reaching 22–26%, as compared to approximately 5% in adults [1]. Thus, it is recommended to perform a fine needle aspiration biopsy (FNAB) in every thyroid lesion found in a child, except for a pure cyst [2]. Contrary to the adult population, the size of the thyroid nodule cannot be considered as an indication for FNAB in children, simply because their thyroid glands are smaller than an adult’s. Thus, even small solid nodules should undergo FNAB. In children, thyroid cancer is often bilateral and multifocal, so none of these features can reduce diagnostic alertness [1,2]. At the time of diagnosis, lymph node metastases are present in most children with thyroid cancer. Hence, precise US examination of lymph nodes should be performed in every child with thyroid lesions and/or palpable suspicious lymph nodes.

In the US, intrathyroidal ectopic thymus (IET) can mimic papillary thyroid carcinoma (PTC), which is the most common thyroid cancer in children. After formation of the definitive thymus, it descends to the upper anterior mediastinum [3,4]. A disturbed process of the migration can result in ectopic thymus location, including an intrathyroidal one. The prevalence of ectopic neck thymus in children was reported as 0.99% [5] to 1.8% [6].

In the US, IETs are usually hypoechoic lesions, with punctate or linear bright internal echoes that look like microcalcifications [7]. In some cases, the lesion margins are irregular. Such features suggest PTC and require precise differential diagnosis [7]. The extrathyroidal ectopic thymic tissue (EET) is most often located very close to the lower pole of one of the thyroid lobes. Its US pattern may strongly suggest the presence of a pathological lymph node [7].

The usefulness of the strain elastography (SE) in the differential diagnosis of IETs was previously demonstrated by our research team [7]. This method evaluates tissue stiffness (elasticity) by measuring the degree of tissue deformation in response to mechanical compression [7,8,9]. The stiffness of the examined lesion must be compared to the adjacent healthy tissue to calculate the difference in relative stiffness. This difference is presented as a strain ratio (SR). This procedure helps to differentiate malignant thyroid lesions—which are stiffer than the surrounding thyroid tissue–from IETs, of which the SR was demonstrated to be low (0.95 to 1.09) [7]. However, SE has important limitations. Firstly, the SR result cannot be considered as absolutely investigator-independent because manual external compression in SE leads to operator-dependent variability. Secondly, the result does not provide quantitative data on the lesion’s stiffness. Therefore, the usefulness of this method is limited to the lesions which stiffness can be compared to the surrounding healthy tissue. Thus, SE is useless in the case of some EETs and suspicious lymph nodes.

Shear wave elastography (SWE) is a further improvement of sonoelastography, providing a two-dimensional distribution map of tissue stiffness and quantitative measurement of the tissue stiffness in Young’s modulus (kPa) and/or shear wave speed (m/s) [10]. Thus, SWE seems to be a great diagnostic tool for both IET and EET neck lesions.

The aim of the study was to demonstrate the usefulness of SWE in the differential diagnosis of ETs and to compare the results of SWE with SE. The additional aim of the study is to present US features of IETs and EETs in the largest group of cytologically confirmed ETs described so far.

## 2. Materials and Methods

### 2.1. Patient Selection and Diagnostic Procedures

Fifty three cytologically confirmed ETs found in 31 children who were referred to our Department because of suspicion of PTC/neoplastic lymph node were included in the study. Among the 53 lesions, 36 were IETs and 17 were EETs. In all patients, laboratory tests were performed, including thyrotropin (TSH), free thyroxine (FT4), free triiodothyronine (FT3), anti-thyroid peroxidase antibodies (aTPO), anti-thyroglobulin antibodies (aTg), and TSH receptor antibodies (TRAb). All parameters were measured by electrochemiluminescence immunoassay (ECLIA), Cobas e601 analyzer (Roche Diagnostics, Indianapolis, IN, USA). In all the children, US examination was performed in supine position using a 7–14 MHz linear transducer (AplioXG, Toshiba Medical Systems Corp., Tochigi-ken, Japan). Strain elastography was performed in all IET lesions (AplioXG, Toshiba Medical Systems Corp., Japan). Shear wave elastography was performed in all lesions (Aixplorer MACH30, Supersonic Imagine, France). The shear wave stiffness (SWS) and shear wave ratio (SWR) values were measured for thyroid/IET pairs and for normal thymus in transverse plane and for thyroid/EET pairs in longitudinal plane as there was no possibility of simultaneously presenting the EET and corresponding thyroid tissue in a transverse plane. All SWS measurements were recorded in kilopascals. In all patients, FNAB with US guidance was performed under moderate sedation or general anesthesia. Cytological smears were evaluated by the same high-volume pathologist. The presence of only small lymphocytes with scattered epithelioid cells (without macrophages, eosinophils, plasma cells, histiocytes, or other cell types) was considered typical for thymic tissue. The absence of lymphocytes of different stages of differentiation (as well as other cells typical for lymph nodes) allowed differentiation with lymph nodes or other lymphatic tissues. The absence of oncocytic follicular cells and plasma cells allowed exclusion of lymphocytic thyroiditis.

### 2.2. Statistical Analysis

The basic statistical analysis of the collected data was performed using Microsoft Excel statistical functions. Excel was also used to compute Pearson correlation coefficient values *r*. Statistical library scipy.stats for Python was applied to verify the correlation results and to compute the associated *p*-values (*p)*. It was also used for the Mann-Whitney rank test of statistical significance between shear wave stiffness of IET and normal thymus. The *p*-value *p* ≥ 0.05 was considered as statistically not significant.

### 2.3. Bioethical Procedures

In all the cases, written informed consent for all the performed procedures and for the publication of the results were obtained from the patients’ parents. The study was accepted by the Polish Mother’s Memorial Hospital—Research Institute Bioethical Committee, approval code 45/2020.

## 3. Results

### 3.1. Clinical Findings

The mean age of our patients was 6.91 ± 2.44 years, ranging from 3 to 12 years. The female to male ratio was 1:1.38. None of the children had any family history of thyroid carcinoma, nor any medical history of irradiation. None of the children had signs or symptoms of any relevant disease. In all patients, results of tests of thyroid hormones and thyroid antibodies were within the normal ranges (Table 1).

### 3.2. Sonographic Findings

A total number of 53 ectopic thymic tissues were found in 31 patients. Thirty-six lesions were IETs and 17 were EETs. Ultrasound features of the analyzed lesions are presented in Table 2. The size of IETs varied from 4 to 15 mm and the mean largest dimension was 6.64 ± 2.34 mm. The size of EETs ranged from 6 to 23 mm, with the mean largest dimension of 15.59 ± 4.56 mm. Among IETs, 15 lesions were located in the right lobe and 21 in the left lobe; 22 were located in the middle part of the thyroid lobe, 13 in the lower part, and 1 in the upper part of the thyroid lobe. In six children, IET were located bilaterally, while EET were bilateral in two patients (Table 2). Coexistence of EET and IET was found in 8 children. The mean age of children with bilateral IETs was 5.83 years. The girl with the highest number of IETs (7) was 3 years old.

### 3.3. Elastographic Findings

In SWE, the mean SWR of IETs (SWR_IET_) was 0.89 ± 0.21, ranging from 0.5 to 1.2. Thus, the stiffness of IETs was comparable to or lower than the stiffness of the adjacent thyroid tissue (Table 3). The mean SWS of IETs (SWS_IET_) was 7.47 ± 1.93 kPa, while the mean SWS of the adjacent thyroid tissue was 8.66 ± 2.42 kPa (Table 3). No difference between the SWS_IET_ and SWS of the normal thymus (SWS_t_) was found (*p* = 0.236).

**Figure 1:**
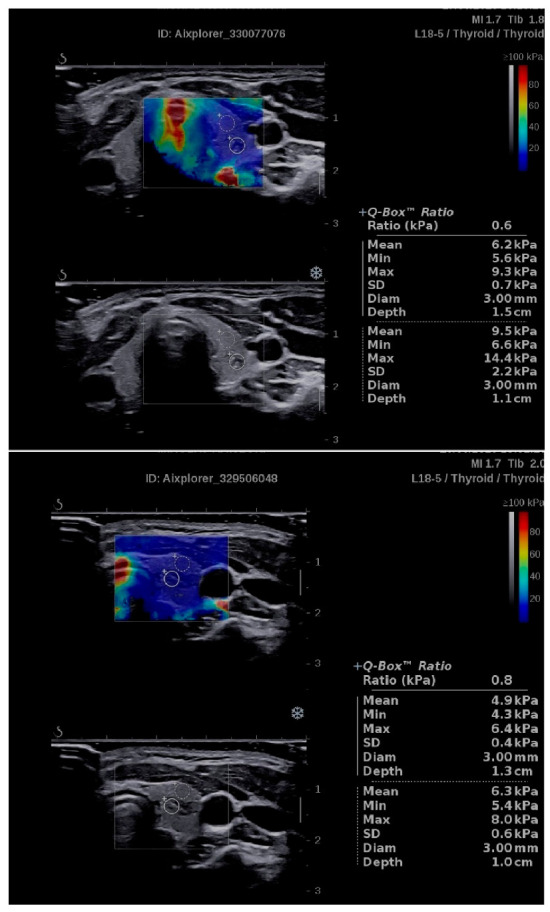
Examples of the application of shear wave elastography (SWE) in intrathyroidal ectopic thymuses (IETs). Quantitative assessments of shear wave stiffness (SWS_IET_) and shear wave ratio (SWR_IET,_ described as “Ratio” in the Figure) allow one to confirm that the stiffness of the analyzed lesion is lower than the surrounding thyroid parenchyma and that the mean values of SWS_IET_ are very low (6.2 and 4.9 kPa).

Examples of SWE of IETs, with quantitative assessments of SWS_IET_ and SWR_IET_, are presented in Figure 1.

**Figure 2:**
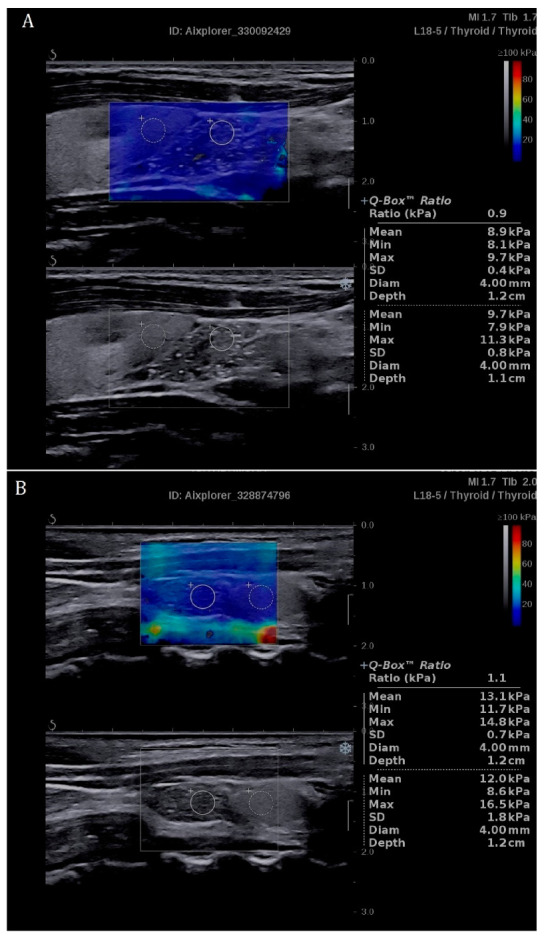
Examples of application of shear wave elastography (SWE) in extrathyroidal ectopic thymuses (EETs). Quantitative assessments of shear wave stiffness (SWS_EET_) and shear wave ratio (SWR_EET,_ described as “Ratio” in the Figure) allow one to confirm that the stiffness of the analyzed lesion is lower (**A**) or similar (**B**) to the surrounding thyroid parenchyma and that the mean values of SWS_IET_ are low in both of the cases—8.9 kPa (**A**) and 13.1 kPa (**B**).

In SE of IET lesions, the SR ranged from 0.5 to 1.2, mean 0.99 ± 0.13 (Table 3), which is comparable to the mean SWR_IET_ result of 0.89 ± 0.21. In SWE of EETs, the comparison with the adjacent thyroid tissue was possible in 15 lesions (longitudinal section) and the mean SWR was 0.84 ± 0.15 (Table 3). The mean SR value in SE was 0.89 ± 0.13. The mean SWS was 11.08 ± 2.57 kPa (Table 1). Examples of SWE of EETs with quantitative assessment of SWS_EET_ and SWR_EET_ are presented in Figure 2.

No statistically significant correlation between patient’s age and SWS_IET_ (*r* = −0.225, *p* = 0.250) or SWS_EET_ (*r* = −0.029, *p* = 0.917) was observed.

**Figure 3:**
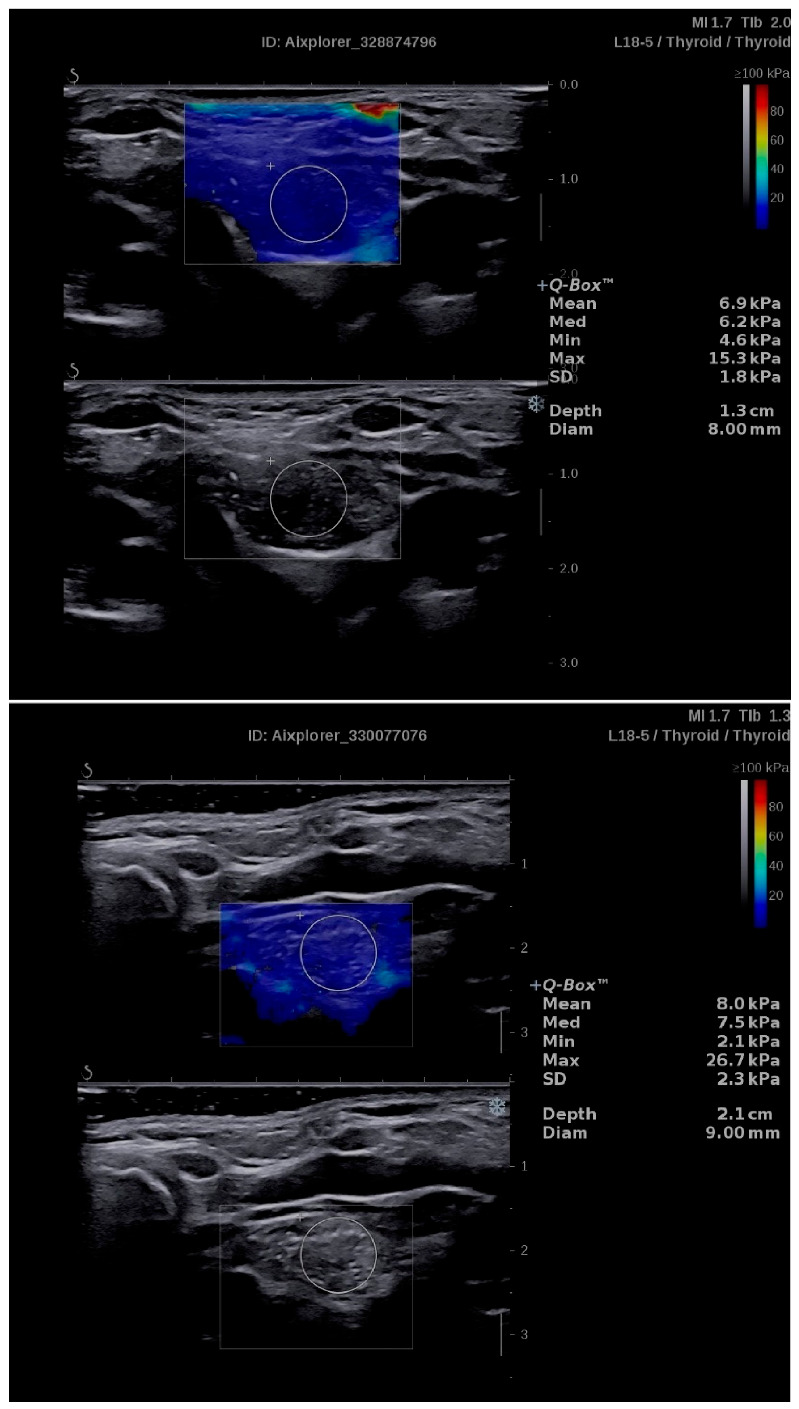
Examples of application of shear wave elastography (SWE) in the evaluation of normal thymus. Quantitative assessments of shear wave stiffness (SWS_t_) reveal low values (6.9 kPa and 8.0 kPa, respectively), which are similar to those of intrathyroidal or extrathyroidal ectopic thymic tissues.

The mean SWS of the normal thymus (SWS_t_) in the analyzed group of children was 6.96 ± 1.58 kPa (Table 3). No statistically significant correlation between SWS_t_ and patient age was observed (*r* = 0.225, *p* = 0.250). Examples of thymus SWE with quantitative assessment of SWS_t_ are presented in Figure 3.

## 4. Discussion

The presence of a pathological lesion in the thyroid or in other cervical locations in children always arouses diagnostic alertness. IETs and EETs are frequently difficult to differentiate from potentially malignant lesions. Ectopic thymic tissues are found in children only and most of the patients are younger than 10 years of age. In this group of patients, FNAB is possible to be performed mainly in sedation or general anesthesia, which requires hospitalization. Moreover, finding a suspicious thyroid/neck lesion in a child generates significant stress for the patient and his/her parents, who need to know promptly whether the lesion may be malignant. For all of these reasons, it is necessary to find an accurate diagnostic method for a quick and simple differentiation between really suspicious lesions that require immediate FNAB and IETs/EETs, which may be observed for some time and in some cases, FNAB may even be avoided, especially if there are some relative contraindications for anesthesia.

We have recently demonstrated the usefulness of SE in the differential diagnosis of IETs [7]. Except for that paper, there is only one other published report on SE application in IETs [11]. To our best knowledge, the current study is the only one in the literature focusing on SWE in ET. Thus, comparison with other authors’ results is not possible.

In the present study, we have confirmed the great usefulness of sonoelastography in ET diagnosis. SWE was demonstrated to be an accurate tool, providing a quantitative value of SWS (kPa) which is independent of any additional manual pressure required in SE. The mean SWS_IET_ and SWS_EET_ were 7.47 ± 1.93 kPa and 11.08 ± 2.57 kPa, respectively. The mean SWS_IET_ value was very similar to the SWS_t_ (6.96 ± 1.58 kPa). The differences in SWS_IET_ and SWS_EET_ and between SWS_EET_ and SWS_t_ result from the fact that SWS_IET_ and SWS_t_ were assessed in the transverse section, while SWS_EET_ were assessed in the longitudinal section. Therefore, SWS_EET_ values should not be directly compared with SWS_IET_ or SWS_t_. The only clinical parameter that can potentially influence SWS of ETs is age as thymus tissue involution and fibrosis progresses with age. However, in our study, neither in IETs/EETs nor in normal thymus was any statistically significant association between SWS and the patients’ age found. The lack of such a correlation probably resulted from the prepubertal status of all our patients. The results of SWR in IETs and in the selected EETs were 0.89 ± 0.21 and 0.84 ± 0.15, respectively. They were similar to the obtained SR values, which were 0.99 ± 0.13 and 0.89 ± 0.13, respectively. The values of SWR and SR observed in the present study confirmed our previous results based on SE, which demonstrated the SR mean value of 1.02 [7]. The only other paper published on this issue reported mean SR values in 22 IETs of 0.99 (ranging from 0.96 to 1.05) [11]. In that group, seven IETs were confirmed cytologically and 15 were diagnosed on the basis of US features. Their results together with ours—both the previous ones and the ones reported in the present paper—proved that the crucial value of sonoelastography in differential diagnosis of ETs is the fact that the stiffness of thymic tissue is lower or similar to the thyroid. This phenomenon is clearly visible on the basis of SWS values (kPa) for ET, which are lower or similar to those of the thyroid. This low stiffness is comparable to that of the normal thymus. On the contrary, the stiffness of malignant lesions is much higher than the stiffness of the adjacent thyroid and the presence of microcalcifications further increases the SWS values [12]. This is of great importance in the differentiation of IET and PTC since PTC is known to be significantly stiffer than thyroid tissue [13]. Chen et al. [14] observed the statistically significant difference between SWS of benign and malignant thyroid lesions, with mean values of 19.2 ± 7.1 kPa and 34.6 ± 14.8 kPa, respectively. The authors demonstrated that the mean SWS cut-off level equal to 24 kPa attained a sensitivity of 78.8% and a specificity of 84.9% [14]. Similar results were presented by Samir et al. [15], who reported a cut-off value for suspicion of malignancy of 22.3 kPa, having a sensitivity, specificity, positive predictive value, and negative predictive value of 82%, 88%, 75%, and 91%, respectively. The highest SWS values obtained in our study for IETs and EETs were 12.7 kPa and 16.6 kPa, respectively. These differences in SWS for malignant and benign thyroid lesions are particularly useful in the case of PTC, which covers over 90% of all thyroid malignancies in children [1]. Follicular cancer, which is often soft in elastography and different in US pattern, is extremely rare in this age group. Therefore, the usefulness of sonoelastography is of particular importance in children because it can be assumed that all lesions resembling IETs in US of which the SWS is similar to or lower than the thyroid tissue, are actually IETs. On the basis of our results and the studies by Chen at al. [14] and Samir et al. [15], we postulate to consider the ET-like lesion suspected of malignancy in the cases with the SWS value greater than 22.3 kPa (the cut-off value proposed by Samir et al. for thyroid nodules [15]).

Bayramoğlu et al. [16] have recently published a paper which provides normative values of SWS_t_ in regard to age and gender. In that study, the mean value of SWS_t_ was 6.76 ± 1.04 kPa, ranging from 4.50 kPa to 10.40 kPa [16]. Our results provide nearly identical values, with the mean SWS_t_ 6.96 ± 1.58, ranging from 4.7 kPa to 10.9 kPa. This high consistency of the SWS_t_ values supports that the obtained results are researcher-independent. Bayramoğlu et al. found significant negative correlations of thymus elasticity and velocity values with age [16]. However, the differences of mean SWS_t_ between individual age groups were found to be statistically insignificant [16]. In our present study, we have not found a significant correlation between SWS_t_ and age, although the obtained value of the Pearson correlation coefficient itself was not much lower. This probably results from the smaller analyzed number of thymuses in our study. Bayramoğlu et al. analyzed SWS values for normal thymuses only, so they could collect a much larger group of children (146 healthy children) [16].

The US-based differential diagnosis between IET and suspicious thyroid nodules is often challenging. Children with IETs are often referred for surgery as hypoechoic nodules with microcalcification-like echoes strongly resemble lesions suspicious of malignancy and cytology frequently reveals a non-diagnostic result (Bethesda category I). The application of SWE together with the detailed knowledge of the US features of ETs can highly facilitate differential diagnosis and can allow one to avoid invasive procedures in a group of children with the values of SWS, SWR, and US features typical for ETs.

Ultrasound characteristics of ETs were reported in only a few large groups of children and the IETs were diagnosed in most of them on the basis of US image only, without cytological evaluation [4,5,11,17,18,19,20,21]. Up to now, the biggest group with cytologically confirmed ETs was described in our previous report and included 16 lesions [7]. In most reports on the US characteristics of ETs, cytological evaluation was not performed despite the small number of lesions [3,20,21]. Such an approach carries the potential risk of misdiagnosis. Therefore, we believe that studies analyzing particular characteristic features of ETs should include lesions with cytologically confirmed ETs only.

Similarly to our previous report [7] and other studies [4,21], in the present study, the analyzed IETs were located mostly in the middle part of the thyroid lobe and less frequently, in the lower part. Having analyzed more than a three times larger number of patients than in our previous report, we found a less frequent occurrence of bilateral IETs (17% vs. 50%) [7]. This observation is more consistent with other authors’ studies [3,4,21], however, on the other hand, the complete absence of bilateral lesions reported in some papers still seems confusing [6,21]. In the US, most of the IET lesions analyzed in our study were oval (58%), fusiform (14%), triangular (14%), or longitudinal (11%) in shape. The predominance of oval and fusiform IETs was also observed in other reports [7]. Oval and triangular shapes also dominated in our EET group. All the ETs were solid, hypoechoic with internal linear or, less often, punctual bright echoes. Linear echoes are easier to differentiate from microcalcifications than the punctual ones. Thus, in the cases with such punctual echoes, the application of SWE can be particularly useful as real microcalcifications substantially increase the SWS value, while the SWS of ETs remains low. Therefore, SWE can serve as a quick and easy tool to differentiate lesions suspicious for PTC or metastatic lymph nodes from ETs.

Most ETs typically have clear margins, but in some cases, the margins can be blurred, which can additionally suggest malignancy. In our present study, as many as 13 (36%) IETs and 2 (12%) EETs had blurred margins. In such US images, SWE can again clarify whether the lesion is actually suspicious.

Blood flow is known to be decreased or absent in ETs [7] and our observations are similar to other authors’ findings [3,4,5,6,17,18,19,20,21]. In 19 (53%) of the IETs and in 7 (41%) of the EETs, no blood flow was detected. However, one should always remember that in a significant portion of PTC, vascularization can be absent and the lack of blood flow in thyroid tumors cannot be considered a benign feature [22]. Thus, once again, the application of SWE can allow us to distinguish ETs and malignancy-suspicious lesions.

It is well known that the US pattern of IETs can mimic PTCs and EETs can resemble metastatic lymph nodes. High volume experience is mandatory to distinguish the suspicious thyroid nodule from IETs in US examination. Punctual bright internal echoes in ETs are actually impossible to differentiate from microcalcifications in US. Moreover, ETs can demonstrate other suspicious US features, such as blurred margins or lack of vascularity. The awareness of typical location and US pattern of ETs is required to start with the US examination of ETs. However, in most cases, the US features do not provide unequivocal differential diagnosis. The introduction of SWE can change the quality of ET imaging as it provides quantitative assessment of the tissue elasticity and, simultaneously, it enables us to compare it with the elasticity of the thyroid. The combination of US pattern and SWE results highly improves the quality of US-based evaluation. In the first step of the diagnostic procedures, this results in the avoidance of unnecessary alert and parents’ stress. In the next steps, with such an accurate diagnostic tool in the hands of an experienced ultrasonographer, this gives a chance to avoid invasive diagnostic procedures or even surgery. A typical US pattern of an ET with low SWS values may empower an experienced specialist to temporarily abandon FNAB, particularly in young children with contraindications to even short-term sedation. Regular US and SWE monitoring is mandatory in such cases.

Moreover, one should remember that rare cases of thymoma, thymic carcinoma, and lymphoblastic lymphoma arising from ET were reported [7,23,24,25,26]. Therefore, the appearance of any suspicious US feature or an increase in SWS with higher SWR always require further diagnosis.

In conclusion, SWE has been demonstrated to be a useful diagnostic method in the differential diagnosis of ETs and malignant lesions. It provides a quantitative assessment of lesion elasticity and calculates the useful SWR value by comparing this elasticity with that of the adjacent thyroid tissue. In the present study, we have provided the SWS and SWR/SR values that should be considered normative for ET. The SWS values for IETs are usually below 10 kPa and the cut-off value of below 22.3 kPa may be considered as typical for benign lesions, including ETs, irrespective of the US plane (transverse or longitudinal), provided that the SWR value is close to 1.0 or lower. Taking into account this SWR criterion is especially important if borderline SWS results are observed. SWE complements the US diagnostics and helps elucidate the suspicious US features of ETs. Therefore, the application of SWE in ET diagnosis allows for more accurate evaluation of ET-like lesions and, in many cases, helps to avoid invasive procedures, simultaneously providing the precise monitoring method based on combined US and SWE evaluation.

## Figures and Tables

**Table 1:** Clinical characteristics of the analyzed group of children.

	Mean ± SD	Range (min–max)
Age (years)	6.91 ± 2.44	3–12
TSH (mIU/L)	2.78 ± 0.95	1.11–4.55
FT4 (ng/dl)	1.30 ± 0.14	1.05–1.56
FT3 (pg/mL)	4.11 ± 0.4	3.06–4.73
anti-Tg (IU/mL)	11.19 ± 2.89	10.0–24.4
anti-TPO (IU/mL)	11.17 ± 5.4	5.0–23.4
TRAb (IU/mL)	0.41 ± 0.2	0.3–0.91

Abbreviations: aTg, thyroglobulin antibodies; aTPO, thyroid peroxidase antibodies; FT4, free thyroxine; FT3, free triiodothyronine; TSH, thyroid stimulating hormone; TRAb, TSH receptor antibodies. Reference ranges: TSH 0.7–5.97 mIU/L for children <6 years, 0.6–4.48 mIU/L for children 6–12 years; FT4 0.97–1.67 ng/dl; FT3 2.53–5.22 pg/mL; aTPO < 34 IU/mL; aTg < 115 IU/mL; TRAb < 1.75 IU/mL.

**Table 2:** Sonographic features of IETs and EETs.

	IET No. (%)	EET No. (%)
Total No. of lesions	36 (100)	17 (100)
Total No. of children with lesions	24 (NA)	15 (NA)
Shape		
• oval • fusiform • triangular • round • rhomboid • longitudinal	21 (58)	6 (35)
	5 (14)	1 (6)
	5 (14)	5 (29)
	1 (3)	1 (6)
	0 (0)	2 (12)
	4 (11)	2 (12)
Margin		
• clear • blurred	23 (64)	15 (88)
	13 (36)	2 (12)
Part of the lobe		NA
• upper • middle • lower	1 (3) 22 (61) 13 (36)	
Side		
• right • left	15 (42)	10 (59)
	21 (58)	7 (41)
No. of patients with bilateral lesions	6 (17)	2 (12)
Mean maximal size ± SD (mm) • Range (mm)	6.64 ± 2.34	15.59 ± 4.56
	4–15	6–23
Maximal number of lesions in a child	7 (NA)	2 (NA)
Bright echo shape		
• punctual • linear	9 (25)	7 (41)
	27 (75)	10 (59)
Vascularization		
• decreased • absent	17 (47)	10 (59)
	19 (53)	7 (41)

Abbreviations: EET, extrathyroidal ectopic thymus; IET, intrathyroidal ectopic thymus; NA, not applicable; SD, standard deviation.

**Table 3:** Sonoelastographic data of IETs, EETs, and normal thymuses.

	IET SWS	Thyroid SWS	IET SWR	IET SR	EET SWS *	Thyroid SWS *	EET SWR *	EET SR *	Thymus SWS
Mean ± SD	7.47	8.66	0.89	0.99	11.08	13.45	0.84	0.89	6.96
	±1.93	±2.42	±0.21	±0.13	±2.57	±4.30	±0.15	±0.13	±1.58
Median	6.9	8.7	0.9	0.9	11.0	12.75	0.85	0.85	6.8
Minimum	3.8	4.7	0.5	0.5	5.9	6.8	0.5	0.71	4.7
Maximum	12.7	15	1.2	1.2	16.6	22.5	1.1	1.16	10.9

* EET data were collected in the longitudinal section. Abbreviations: EET, extrathyroidal ectopic thymus; IET, intrathyroidal ectopic thymus; SD, standard deviation; SR, strain ratio; SWR, shear wave ratio; SWS, shear wave stiffness.

## Abbreviations

EET	extrathyroidal ectopic thymic tissue;
ET	ectopic thymus;
FNAB	fine needle aspiration biopsy
IET	intrathyroidal ectopic thymus;
PD	power Doppler;
PTC	papillary thyroid carcinoma;
SE	strain elastography
SR	strain ratio;
SWE	shear wave elastography
SWS	shear wave stiffness
SWR	shear wave ratio
US	ultrasound.
